# Preliminary exploration of deep learning-assisted recognition of superior labrum anterior and posterior lesions in shoulder MR arthrography

**DOI:** 10.1007/s00264-023-05987-4

**Published:** 2023-09-20

**Authors:** Ming Ni, Lixiang Gao, Wen Chen, Qiang Zhao, Yuqing Zhao, Chenyu Jiang, Huishu Yuan

**Affiliations:** Department of Radiology, Peking University Third Hospital, 49 Huayuan North Road, Haidian District Beijing, People’s Republic of China

**Keywords:** Shoulder, Deep learning, Arthrography, Superior labrum from anterior to posterior injuries, Artificial intelligence

## Abstract

**Purpose:**

MR arthrography (MRA) is the most accurate method for preoperatively diagnosing superior labrum anterior–posterior (SLAP) lesions, but diagnostic results can vary considerably due to factors such as experience. In this study, deep learning was used to facilitate the preliminary identification of SLAP lesions and compared with radiologists of different seniority.

**Methods:**

MRA data from 636 patients were retrospectively collected, and all patients were classified as having/not having SLAP lesions according to shoulder arthroscopy. The SLAP-Net model was built and tested on 514 patients (dataset 1) and independently tested on data from two other MRI devices (122 patients, dataset 2). Manual diagnosis was performed by three radiologists with different seniority levels and compared with SLAP-Net outputs. Model performance was evaluated by the receiver operating characteristic (ROC) curve, area under the ROC curve (AUC), etc. McNemar’s test was used to compare performance among models and between radiologists’ models. The intraclass correlation coefficient (ICC) was used to assess the radiologists’ reliability. *p* < 0.05 was considered statistically significant.

**Results:**

SLAP-Net had AUC = 0.98 and accuracy = 0.96 for classification in dataset 1 and AUC = 0.92 and accuracy = 0.85 in dataset 2. In dataset 1, SLAP-Net had diagnostic performance similar to that of senior radiologists (*p* = 0.055) but higher than that of early- and mid-career radiologists (*p* = 0.025 and 0.011). In dataset 2, SLAP-Net had similar diagnostic performance to radiologists of all three seniority levels (*p* = 0.468, 0.289, and 0.495, respectively).

**Conclusions:**

Deep learning can be used to identify SLAP lesions upon initial MR arthrography examination. SLAP-Net performs comparably to senior radiologists.

## Introduction

Superior labrum anterior–posterior (SLAP) lesions were first proposed by Andrews et al. in 1985 and classified by Snyder et al. in 1990 [1, 2]. SLAP lesions are anterior-to-posterior lesions of the superior glenoid labrum and may involve the attachment of the tendon of the long head of the biceps brachii [3]. With the development of arthroscopic repair technology, the number of patients with SLAP lesions who undergo arthroscopic repair is also increasing [4, 5]. Therefore, it is crucial to accurately identify patients with SLAP lesions before surgery, which can reduce the delay caused by diagnostic errors.

The clinical diagnosis of SLAP lesions is challenging [6, 7]. MRI is widely used in preoperatively evaluating SLAP lesions due to its good soft tissue resolution. However, study results have shown that the accuracy, sensitivity, and specificity of conventional MRI in diagnosing SLAP lesions are not high [8, 9]. MR arthrography (MRA) is currently considered the most accurate method for the preoperative assessment of SLAP lesions, with a higher average sensitivity (80.4%) and average specificity (90.7%) than conventional MRI [10]. Researchers have found that the diagnosis of SLAP lesions is mainly affected by the experience of radiologists [11], that overdiagnosis may occur [9], and that the false positive rate of diagnosis is high due to the existence of anatomical variation [6].

With the development of hardware and the improvement of algorithms, artificial intelligence research, especially in the area of deep learning, has brought about rapid progress in imaging [12]. Deep learning aims to simulate the structure and function of the human brain’s neural network to learn and extract complex features and patterns from data. The core idea is to use a multilayered neural network to build a cascaded feature extraction and transformation process, where each layer performs some transformations on the input data, gradually mapping the data to a higher-level abstract representation. Deep learning can efficiently and stably assist radiologists in diagnosing and classifying various diseases [13]. Recent research results show that deep learning can assist in identifying and classifying various sports injuries, reaching or even exceeding the performance of senior musculoskeletal (MSK) radiologists [14, 15]. Nevertheless, deep learning research on sports injury diseases is still limited, and many common sports injuries remain to be explored.

This study aims to preliminarily explore the feasibility of deep learning in shoulder MRA for SLAP lesions to assist radiologists in identifying SLAP lesions more accurately, reduce diagnostic errors caused by differences in radiologist experience, and provide more accurate information for clinical practice.

## Materials and methods

The institutional review board of our hospital and the medical science ethics committee approved this retrospective study, and the requirement for informed consent was waived (ethical approval number: IRB00006761-M2020458).

### Patients

**Fig. 1:**
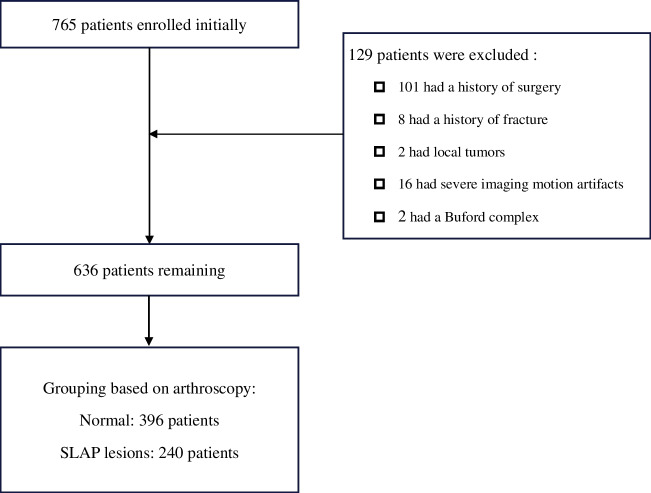
Flowchart showing the baseline patient characteristics

The clinical and imaging data of patients (including adults and adolescents) undergoing shoulder MRA in our hospital were collected retrospectively. The inclusion criterion was MRA examination within ten days before arthroscopic surgery. The exclusion criteria were as follows: (1) poor image quality with many motion artifacts (assessed by M.N., with eight years of experience); (2) previous history of shoulder surgery, tumor, fracture, autoimmune disease, or infection; and (3) Buford complex. From January 2013 to August 2022, 765 patients underwent an MRA examination, and 636 were ultimately included in the study according to the above criteria, as shown in Fig. 1.

All patients underwent shoulder arthroscopic surgery by specialized surgeons in the Sports Medicine Center of our hospital, and the presence or absence of SLAP lesions (class 0: normal; class 1: SLAP lesions) was determined according to the arthroscopy results. The normal patients comprised patients with other types of labral injuries (such as Bankart injuries), rotator cuff tears, and adhesive capsulitis as well as patients with suspected but later disproven SLAP injuries or anatomical variations on conventional MRI.

### MR arthrography

Shoulder arthrography was performed by two trained radiologists (W.C., with 20 years of experience, and Y.Z., with 10 years of experience). The contrast medium was made by mixing 0.25 ml gadopentetic acid (Gd-DTPA), 5 ml iodine contrast medium, 5 ml lidocaine (0.02 g/ml), and 5 ml normal saline; the contrast medium was injected by an anterior approach with a 20-G syringe. For injection into the glenohumeral joint space, the routine injection dose ranged from 15 to 18 ml; the patient was supine during injection, with the upper arm slightly externally rotated, and the palm and elbow fossa were facing upward. After injection of 1–2 ml of contrast medium, X-ray imaging was performed to confirm that the contrast medium had successfully entered the joint space, and MRA examination was completed within 30 min after the injection of the contrast medium.

MRA was performed with a Discovery 750 W Silent (3.0 T GE Medical Systems, Waukesha, WI, USA), a Magnetom Trio (3.0 T, Siemens Healthcare, Erlangen, Germany), and a uMR 770 (3.0 T, United-Imaging Healthcare, Shanghai, China) using an ultrasoft coil dedicated to the shoulder joint for scanning. The scans were axial, oblique coronal (OCOR), and oblique sagittal (OSAG) fat-saturation T1-weighted fast spin‒echo (T1-FSE-FS) and OSAG T1-weighted fast spin‒echo (T1-FSE) sequences. The deep learning model in this study used only axial and OCOR T1-FSE-FS. The corresponding parameters were as follows: axial T1-FSE-FS, repetition time (TR) of 626–691 ms, echo time (TE) of 8.1–9.7 ms, flip angle of 111°–120°, field of view (FOV) of 160 × 160, slice thickness of 3 mm, spacing between slices of 0.3–0.5 mm and number of excitations (NEX) 1.5–2, OCOR T1-FSE-FS, TR of 554–576 ms, TE of 9.7–13.7 ms, flip angle of 111°–120°, FOV 160 × 160, slice thickness of 3 mm, spacing between slices of 0.6 mm, and NEX 1.2–2.

### Region of interest

**Fig. 2:**
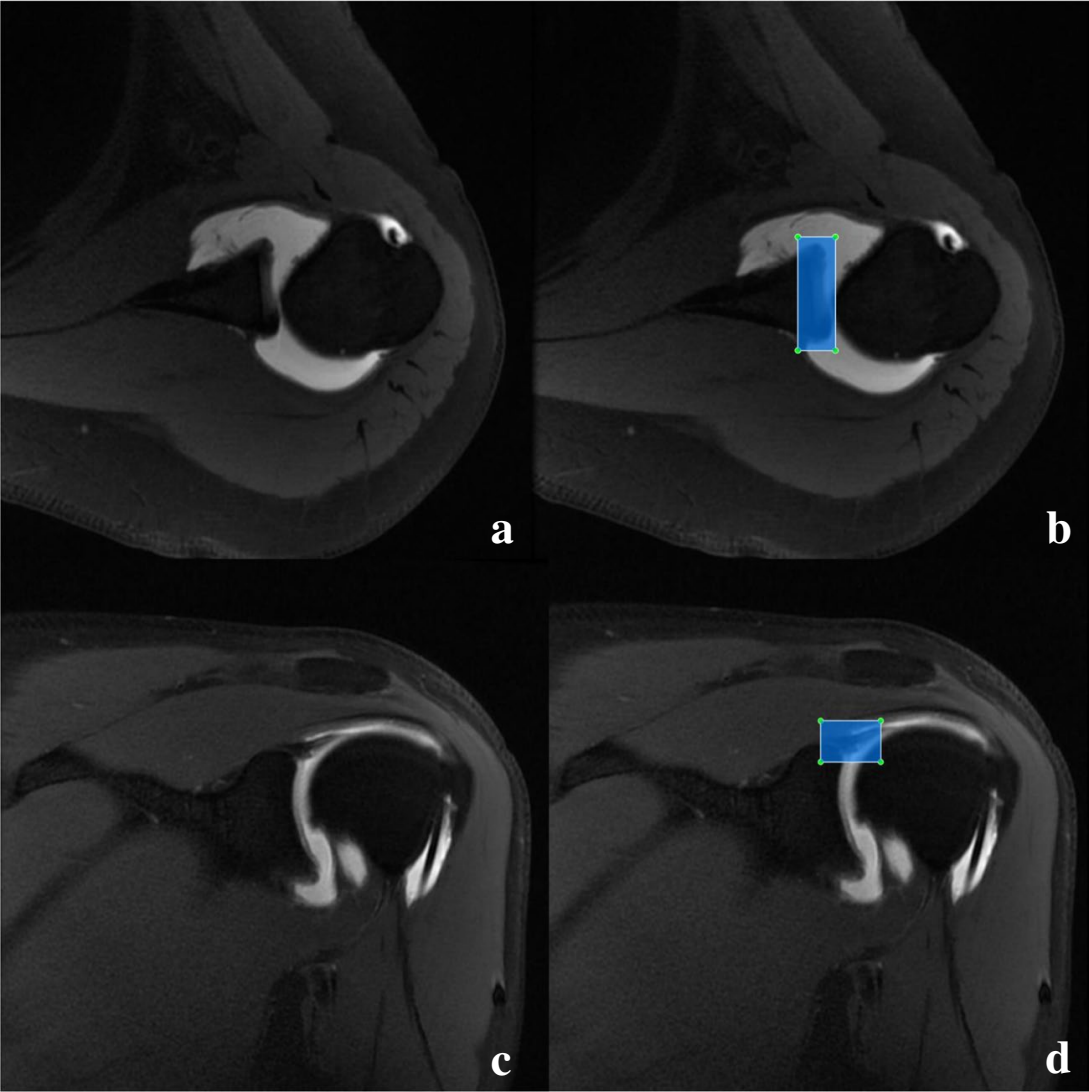
Examples of the manual drawing of ROIs. The figure shows the axial and OCOR T1-FSE-FS images, where panels **a** and **c** are the original images, respectively, and panels **b** and **d** are the corresponding bounding boxes. All ROIs were delineated to maximize the inclusion of labral structures while minimizing other additional structures

Two radiologists (W.C. and M.N.) with different levels of seniority delineated all Discovery 750 W Silent images using Python-based labeling software (https://github.com/tzutalin/labelImg) and drew the region of interest (ROI) in the form of a bounding box. After the initial drawing by M.N., W.C. adjusted the result of his drawing to ensure the accuracy of the ROI. In the axial and OCOR T1-FSE-FS images, the ROI was delineated layer by layer to ensure that only the upper labrum structure was included, minimizing the inclusion of other structures. Examples of ROI delineation are shown in Fig. 2.

### Deep learning workflow

**Fig. 3:**
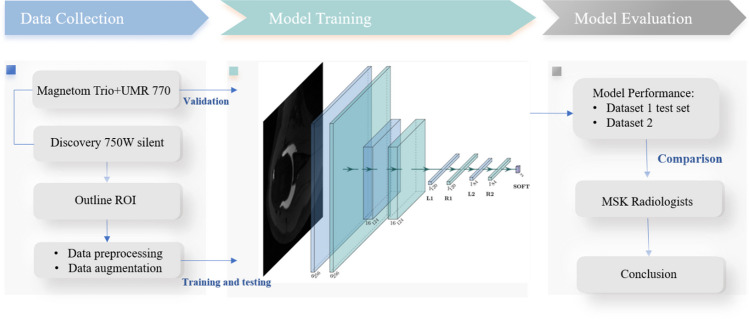
Flowchart of the study process. The study was divided into three parts: data collection, model training, and model evaluation. The data collection stage included MRA acquisition, ROI outlining, and data preprocessing, and the model evaluation stage consisted of verifying the effectiveness of the model based on datasets 1 and 2 and comparing the model with radiologists of different seniority levels

The deep learning models were trained using an NVIDIA Tesla 10 × (32 GB video memory, NVIDIA, Santa Clara, California, USA) and an Intel(R) Xeon(R) Gold 5215 CPU (Intel, Santa Clara, California, USA). A flow chart of the study is shown in Fig. 3.

#### Preprocessing

Image preprocessing can convert images into a form more suitable for machine analysis and processing, eliminate the influence of dimensions between different image features, highlight meaningful information for machine analysis, and suppress irrelevant information to improve the use value of images without changing the image information within. In this study, image preprocessing was performed through normalization:

$$\mathrm{Normalization}=\frac{{x}_{i}-{x}_{\mathrm{min}}}{{x}_{\mathrm{max}}-{x}_{\mathrm{min}}}$$

$$(x\;:\;\mathrm{gray}\;\mathrm{value}\;\mathrm{corresponding}\;\mathrm{to}\;\mathrm{any}\;\mathrm{pixel})$$

Due to the small number of patients in this study and to improve the model’s generalization, data augmentation was performed by randomly changing the image brightness and adding Gaussian noise. Changing the image brightness aims to imitate the adjustment of the image window width/window level when images are observed in daily work, while randomly adding Gaussian noise imitates images of different quality levels acquired with different equipment and parameters. Finally, all images were randomly shuffled before model training.

#### Recognition of SLAP lesions

In this study, we developed SLAP-Net based on deep learning to identify SLAP lesions in MRA images. The network comprises nine layers: two convolutional layers, two batch normalization layers, two maximum pooling layers, and three linear layers. Figure 3 (“Model Training” column) shows the model structure diagram. SLAP-Net uses the Adam optimizer, the loss function is weighted cross-entropy loss (the weights of classes 0 and 1 are 1 and 1.6), and the learning rate is dynamically adjusted through the cosine annealing strategy. We used all the images from the Discovery 750 W Silent (referred to as dataset 1), divided into a training set, a verification validation set and a test set at an 8:1:1 ratio, to train and test the SLAP-Net model; we then used all images from the Magnetom Trio and UMR 770 (dataset 2, not involved in model building) for independent testing. During model training, the axial and oblique coronal T1-FSE-FS images with ROIs were combined as input, and the category with the highest frequency was output as the final diagnosis result for the patient.

#### Radiologist evaluations

Three radiologists with different levels of seniority (radiologist 1, L.G., with 15 years of experience; radiologist 2, Y.Z.; and radiologist 3, C.J., with 7 years of experience) independently compared the test set in dataset 1 and all patients in dataset 2. The final result was obtained through a comprehensive judgment of all sequences in the diagnosis. All radiologists had participated in the shoulder joint MRI diagnosis training course in our hospital and completed the course assessment; we expect that this uniform training eliminated the differences in disease understanding caused by different concepts and other reasons.

### Statistical analysis

The statistical and deep learning analyses were performed using Python (version 3.6.0; Python Software Foundation, Fredericksburg, VA, USA) software, and data processing was carried out with the PyTorch (version 1.1.0) framework based on dataflow programming. Model performance was assessed by receiver operating characteristic (ROC) curve analysis and evaluated in terms of accuracy, sensitivity, specificity, positive predictive value (PPV), negative predictive value (NPV), and area under the ROC curve (AUC). The intraclass correlation coefficient (ICC) was used to assess the reliability of the diagnoses between the MSK radiologists. McNemar’s test was used to compare performance between the radiologists and SLAP-Net models. *p* < 0.05 indicated a statistically significant result.

## Results

Among the 636 patients in this study, 514 patients were scanned with a Discovery 750 W Silent, including 318 patients in class 0 (sex: 259 male; 59 female; age: 30.2 ± 10.6) and 196 patients in class 1 (sex: 158 male; 38 female; age: 31.8 ± 9.6). The data of a total of 122 patients were collected with a Magnetom Trio (*n* = 102) and a UMR 770 (*n* = 20); among these patients, there were 78 in class 0 (sex: 55 male; 23 female; age: 30.8 ± 12.1) and 44 in class 1 (sex: 22 male; 22 female; age: 31.8 ± 9.6).

**Fig. 4:**
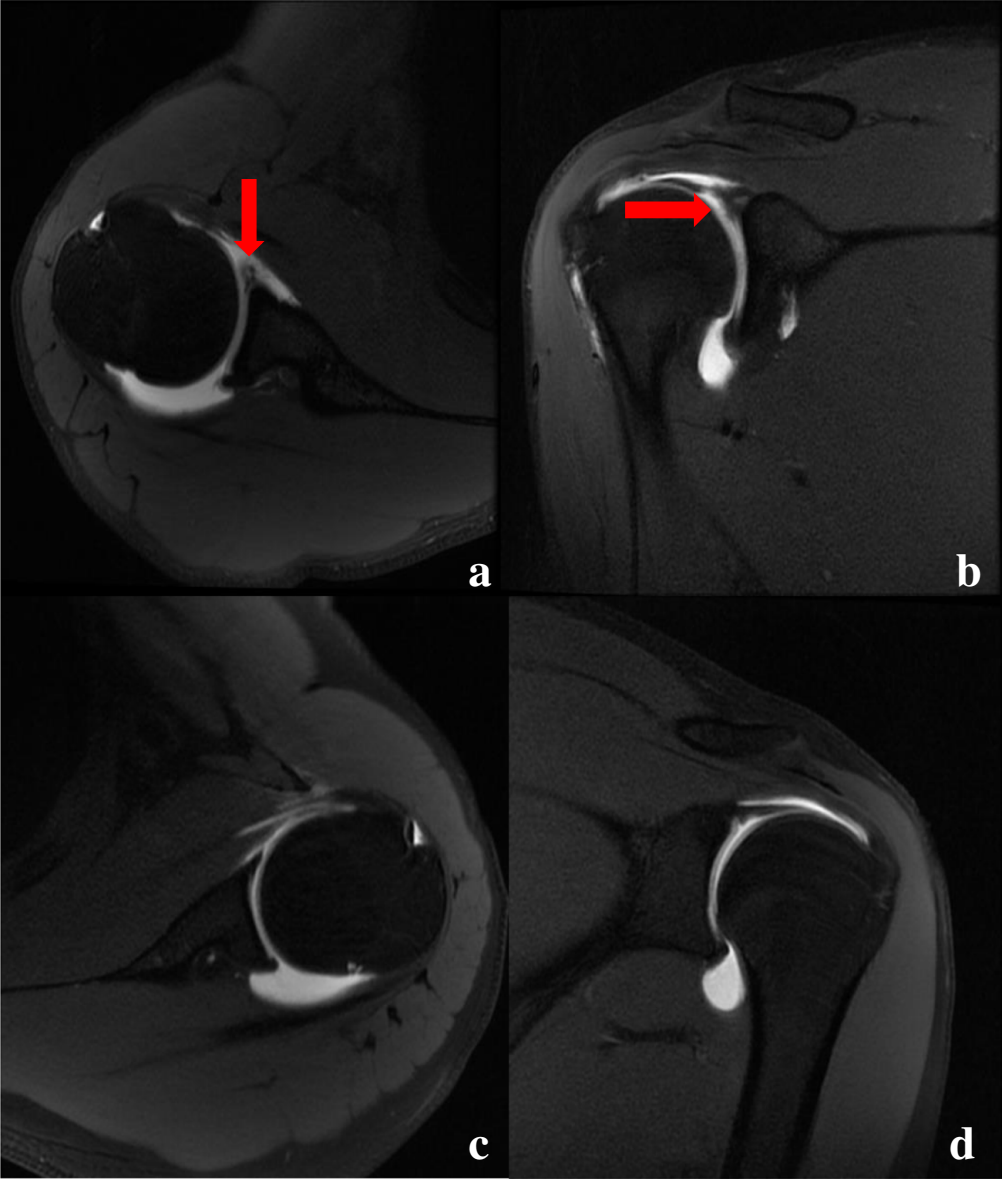
Examples of manually diagnosed and SLAP-Net-diagnosed errors. Panels **a** and **b** show a 25-year-old male patient diagnosed with SLAP type 2 by arthroscopic surgery but diagnosed as normal by radiologist 3; the model diagnosis was correct. The red arrows indicate SLAP lesions. Panels **c** and **d** show a 21-year-old male patient. The results of arthroscopic surgery showed no SLAP lesions, but SLAP-Net diagnosed SLAP lesions; the manual diagnosis was correct

**Fig. 5:**
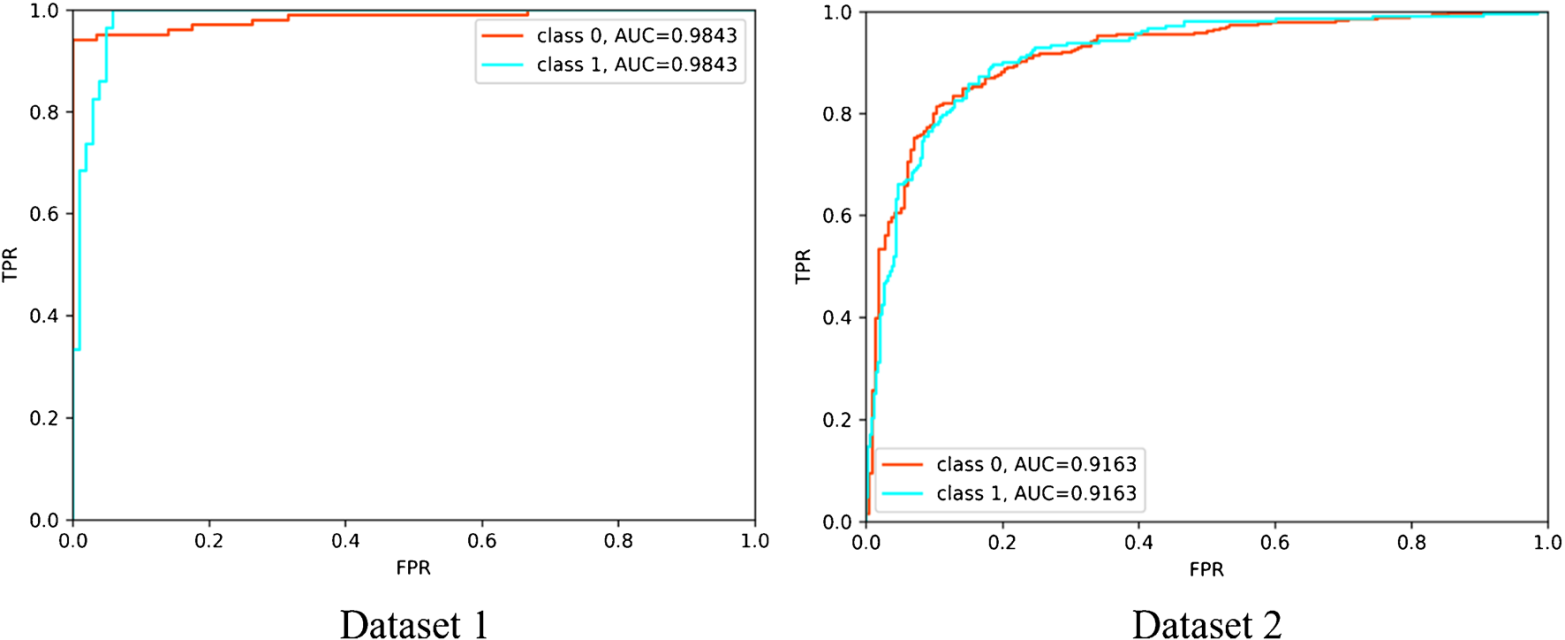
The classification ROC curves of SLAP-Net on datasets 1 and 2. The classification AUC of SLAP-Net on dataset 1 is 0.98, and its classification AUC on dataset 2 is 0.92

The SLAP-Net established in this study had an AUC of 0.98 (95% CI: 0.945–1.000) for identifying classes 0 and 1 on the test set of dataset 1, with an accuracy of 0.96, and its AUC for identifying classes 0 and 1 on dataset 2 was 0.92 (95% CI: 0.857–1.000), with an accuracy of 0.85. The agreement of three MSK radiologists with different seniority levels for manual diagnosis on the test set of dataset 1 was 0.996 (95% CI: 0.971–1.000), and the agreement of manual diagnosis on dataset 2 was 0.995 (95% CI: 0.964–1.000). The accuracy levels of class 0 and class 1 identification by radiologists 1, 2, and 3 on the test set of dataset 1 were 0.85, 0.83, and 0.81, respectively. SLAP-Net had similar diagnostic performance to radiologist 1 (*p* = 0.055) and outperformed radiologists 2 and 3 (*p* = 0.025 and 0.011). On dataset 2, the accuracy levels of these three radiologists in identifying classes 0 and 1 were 0.85, 0.86, and 0.81, respectively. The diagnostic performance of SLAP-Net was similar to that of the three radiologists (*p* = 0.468, 0.289 and 0.495), as shown in Table 1. Examples of errors made by the model and manual diagnosis are shown in Fig. 4, and the ROC curve of the model is shown in Fig. 5.

**Table 1:** The diagnostic effect of the SLAP-Net model and three radiologists with different seniority levels on datasets 1 and 2 and a comparison between the model and manual diagnosis

	Dataset	Group	AUC	Accuracy	Sensitivity	Specificity	PPV	NPV	*χ*^2^	*p* value*
SLAP-Net	Dataset 1**	Class 0	0.98	0.96	0.94	1.00	1.00	0.91	/	/
		Class 1	0.98	0.96	1.00	0.94	0.91	1.00	/	/
	Dataset 2**	Class 0	0.92	0.85	0.90	0.76	0.86	0.84	/	/
		Class 1	0.92	0.85	0.76	0.90	0.84	0.86	/	/
Radiologist 1 (15 years of experience)	Dataset 1	Class 0	/	0.85	0.91	0.76	0.86	0.84	3.68	0.055
		Class 1	/	0.85	0.76	0.91	0.84	0.86		
	Dataset 2	Class 0	/	0.85	0.86	0.84	0.91	0.77	0.53	0.468
		Class 1	/	0.85	0.84	0.86	0.77	0.91		
Radiologist 2 (10 years of experience)	Dataset 1	Class 0	/	0.83	0.81	0.85	0.90	0.74	5.04	0.025
		Class 1	/	0.83	0.85	0.81	0.74	0.90		
	Dataset 2	Class 0	/	0.86	0.89	0.82	0.90	0.80	1.13	0.289
		Class 1	/	0.86	0.82	0.89	0.80	0.90		
Radiologist 3 (7 years of experience)	Dataset 1	Class 0	/	0.81	0.78	0.85	0.89	0.71	6.50	0.011
		Class 1	/	0.81	0.85	0.78	0.71	0.89		
	Dataset 2	Class 0	/	0.81	0.86	0.73	0.85	0.74	0.47	0.495
		Class 1	/	0.81	0.73	0.86	0.74	0.85		

*AUC* area under the receiver operating characteristic curve, *PPV* positive predictive value, *NPV* negative predictive value, *χ*^2^ the chi-square value calculated by McNemar’s test

*The results of comparing different radiologists with SLAP-Net on dataset 1 and dataset 2; McNemar’s test was used for the statistical analysis

**Dataset 1 contains 52 test patients, and dataset 2 is an independent test set that contains 122 patients

## Discussion

In this study, we aimed to develop an MRA-based SLAP-Net model and preliminarily explore the feasibility of deep learning for identifying shoulder joint SLAP lesions to reduce the overlooking or over diagnosis of lesions and provide more accurate disease information for clinical practice. The study results show that SLAP-Net has good diagnostic performance in identifying SLAP lesions in images from different devices, reaching the same level of performance as a senior MSK radiologist.

SLAP lesions are an important cause of shoulder pain and instability [16, 17]. Although various physical examinations can be used to detect SLAP lesions, the diagnostic value of these tests is inconsistent, the specificity is low [18–20], and the clinical diagnosis of SLAP lesions is difficult due to the possibility of multiple lesions in the shoulder joint. Therefore, imaging has irreplaceable value in the preoperative identification and evaluation of SLAP lesions. Currently, MRA is considered the preferred method for the preoperative diagnosis of SLAP lesions [21]. Nevertheless, due to anatomical variations of the labrum, complex structures around the upper labrum, radiologists’ inexperience and fatigue, and other factors, the diagnosis of SLAP lesions varies [8]. There are reports that the sensitivity of SLAP lesion diagnosis by MRA is between 0.60 and 0.90, the specificity is between 0.50 and 0.98, and there is large variability between studies of different physicians and institutions [8, 22–24]. Therefore, developing a deep learning method with high stability and high diagnostic efficiency for identifying SLAP lesions will help inexperienced radiologists diagnose SLAP lesions more effectively and reduce patient treatment delays caused by reporting errors.

The treatment of SLAP lesions is still controversial [25]. However, in any case, reasonable intervention should be carried out for SLAP lesions to prevent continuous progression from causing a more substantial impact on patients’ lives. Therefore, it is vital to initially identify which patients have SLAP lesions to avoid delays in diagnosis due to diagnostic errors. However, because MRA examinations are rarely carried out in some medical institutions, many radiologists may lack relevant diagnostic experience, resulting in inaccurate diagnoses. The SLAP-Net model developed in this study can assist these radiologists in diagnosis and is expected to improve diagnostic accuracy, sensitivity, and specificity.

In this study, we used the images in dataset 1 to build and test the SLAP-Net model and used images from the other two devices (dataset 2) to conduct independent tests to verify the generality of the model. The results showed that the model achieved excellent diagnostic performance on dataset 1. Even though the model’s performance on dataset 2 was reduced, it still reached the level of an experienced MSK radiologist, which demonstrates that the model has good general performance. However, due to the lack of an out-of-group test set, a more accurate assessment of generality requires further research in the future. The performance of the SLAP-Net model developed in this study is similar to that of senior MSK radiologists, implying that the model has both good stability and good diagnostic performance.

### Limitations

(1) In this study, we only identified SLAP lesions preliminarily and did not conduct a more detailed classification because only type II SLAP lesions were common in the dataset; the other types were relatively scarce, which may have made for insufficient sample sizes of those. The imbalance between the numbers would lead to poor model performance or even failure to converge. We will conduct further related research after collecting enough data in the future. (2) Our study did not use conventional MRI data as input because MRA is the most accurate preoperative examination method for diagnosing SLAP lesions. In the future, we will also collect data with conventional MRI and conduct related research. (3) This study lacks an out-of-group test set and only uses different devices for verification. Because no suitable dataset has been found thus far, this work will be done in the future. (4) The training, verification, and testing of SLAP-Net were performed only on data from 3.0-T equipment, and no related research has been conducted on data from 1.5-T equipment.

## Conclusion

Our study demonstrates that SLAP-Net has good diagnostic performance in identifying SLAP lesions, and the performance of this model is comparable to that of senior radiologists. Therefore, the SLAP-Net model has the potential to help radiologists and clinicians identify SLAP lesions more accurately before surgery.

## Data Availability

All imaging and clinical data are stored in the database of the Radiology Department of Peking University Third Hospital. Please get in touch with Peking University Third Hospital for data acquisition.

## Abbreviations

MRA: MR arthrography
SLAP lesions: Superior labrum anterior and posterior lesions
ROC curve: Receiver operating characteristic curve
AUC: Area under the ROC curve
ICC: Intraclass correlation coefficient
Gd-DTPA: Gadopentetic acid
MSK: Musculoskeletal
ROI: Region of interest
OCOR: Oblique coronal
OSAG: Oblique sagittal
T1-FSE-FS: Fat-saturation T1-weighted fast spin‒echo

## Code availability

The SLAP-Net code is detailed at https://github.com/sweethearts94/SLAP-Lesions.
